# Clinical and functional outcomes of rehabilitation strategies following arthroscopic repair of chronic isolated peripheral TFCC tears: A scoping review

**DOI:** 10.1016/j.jor.2025.06.028

**Published:** 2025-07-05

**Authors:** Deepthi Paraj, Ashwath M. Acharya, Anil K. Bhat

**Affiliations:** Department of Hand Surgery, Kasturba Medical College, Manipal, Manipal Academy of Higher Education (MAHE), Manipal, Karnataka, India

**Keywords:** Triangular fibrocartilage complex, Rehabilitation, TFCC tears, Palmer type 1 tear, DRUJ instability, Arthroscopic

## Abstract

This scoping review aims to evaluate the existing literature regarding the types of Triangular Fibrocartilage Complex (TFCC) tears that are amenable to repair, the prevalent rehabilitation protocols, and the documented measurable outcomes across various populations. The review was conducted as per JBI methodology and reported as per PRISMA-ScR guidelines. A total of 35 studies published between 1996 and 2024 were included. Palmer Type 1B was the most frequently operated type of tear. The tears were repaired using various arthroscopic-assisted techniques like capsular repair with polydiaxone sutures(PDS) with or without K-wire fixation, transosseous sutures, and ligament-specific repair. The duration of complete immobilization following repair varied significantly among the elbow, forearm, and wrist joints, with six weeks being the most common immobilization period. Patients on average regained 85 % of grip strength, with 87 % successfully returning to their pre-injury activity levels. The scoping review highlights the variability in rehabilitation practices following TFCC repair and the assessment of outcomes, emphasizing the need for level 1 evidence studies to standardize postoperative protocols.

## 1 Introduction

Triangular fibrocartilage complex (TFCC) tears are a frequent source of ulnar-sided wrist pain,^1^ and are identified in around 36–40 % of patients undergoing diagnostic arthroscopy.^2^ Peripheral TFCC tears that fail to respond to conservative management compel surgical intervention, which can be performed through open or arthroscopic approaches.

Along with the TFCC repairs, complete recovery necessitates a carefully graded rehabilitation process that includes phases of immobilization, gradual introduction of a range of motion exercises, and strengthening exercises of secondary wrist stabilizer muscles. This comprehensive approach is essential for enhancing the stability of the distal radio-ulnar joint and ensuring effective stress distribution, which helps prevent re-rupture or further injury to the repaired ligament. Although many studies advocate this rehabilitation protocol, there remains no consensus regarding the optimal duration of immobilization, the timing for initiating a range of motion exercises, or the appropriate phase for strengthening exercises following the repair.

In this scoping review, we aim to systematically analyze the types of TFCC tears that are amenable to surgical repairs by arthroscopic techniques, prevalence of the rehabilitation protocols implemented following repair, clinical and functional outcomes, and return-to-work rates of patients following these interventions.

## 2 Review questions

1. What are the TFCC tears amenable to arthroscopic repair?
2. What are the prevalent immobilization methods, duration of immobilization, and the limb's position during immobilization, following TFCC repair?
3. What are the functional and clinical outcome scores commonly used to report?
4. What are the complications commonly associated with the repair and/or rehabilitation?
5. When do the patients return to work following the surgery?

## 3 Methods

### 3.1 Protocol and registration

This study followed the JBI methodology for the scoping review^3^ with a peer-reviewed protocol and reported according to the Preferred Reporting Items for Systematic Reviews and Meta-Analyses extension for Scoping Reviews (PRISMA-ScR) checklist.^4^

### 3.2 Eligibility criteria

#### 3.2.1 Population

We assessed all scholarly articles in English literature related to the primary repair of post-traumatic chronic isolated tears since the inception of TFCC repairs. The review included studies representing a diverse population across various age groups and occupations. All tears that were repaired using arthroscopic techniques were considered. Furthermore, all studies that included a rehabilitation program specifically designed for implementation under the supervision of a qualified therapist within a clinical environment and which could subsequently be maintained by patients in their home settings were considered. The review also assessed the clinical and functional outcome scores utilized to document outcome measures.

Studies reporting TFCC repairs associated with wrist fractures, dislocations, other ligament injuries, joint instability, and additional procedures (e.g., ulnar shortening osteotomy), studies with incomplete rehabilitation or without clinical or functional outcomes were excluded.

#### 3.2.2 Concept

The scope of this study was to analyze the rehabilitation protocols following TFCC repairs by noting the type of tear, mean duration between injury to TFCC repair, the mean postoperative follow-up period, the period of immobilization, the position of the limb and the number of joints immobilized during the immobilization period, the type and duration of use of removable splints, the commencement of wrist, forearm and elbow mobilization and strengthening exercises if any and the clinical and functional outcomes used to report the outcome-measures were documented. The time taken for the patients to return to their pre-injury activities, the percentage of the study subjects who returned to their activities, and complications associated with the surgery and/or the rehabilitation were also considered.

#### 3.2.3 Context

This scoping review will consider papers focusing on interventions in any geographical location.

### 3.3 Information sources

All papers related to experimental and quasi-experimental study designs were evaluated, including randomized controlled trials (RCTs), non-randomized controlled trials, before-and-after studies, and interrupted time series. In addition, all descriptive cross-sectional and analytical observational studies, including prospective and retrospective cohort studies, case-control studies, and analytical cross-sectional studies, were also included.

Case studies with a sample size of less than five patients, conference abstracts, opinion pieces, letters to the editor, book chapters, and magazine or newspaper articles were excluded.

### 3.4 Search

**Fig. 1:**
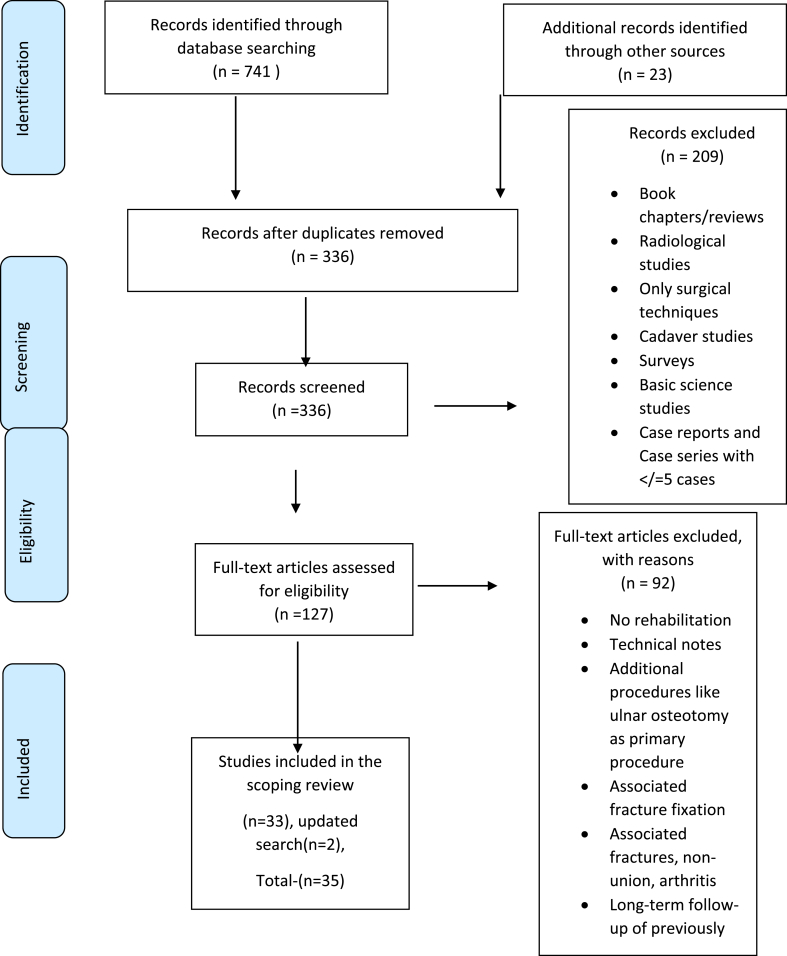
PRISMA flowchart depicting the search results, source selection, and inclusion process.

An initial limited search of PubMed(MEDLINE) and CINAHL was undertaken to identify articles on the topic. The text words in the titles and abstracts of relevant articles and the index terms used to describe the articles were used to develop a complete search strategy for MEDLINE (PubMed; Appendix I – supplementary data). The search strategy, including all identified keywords and index terms, were adapted to each included database and information source. The reference list of all included sources of evidence was screened for additional studies, and a journal search was undertaken to locate further studies. The key terms used for the search were ((TFCC OR TRIANGULAR FIBRO∗ OR TRIANGULAR FIBROCARTILAGE COMPLEX) AND (REPAIR)) AND (EXERCISE OR REHABILITATION OR MOBILIZATION OR IMMOBILIZATION OR THERAPY). The preliminary search was completed by 23rd February 2024, as shown in Fig. 1, and an additional search was conducted on 6th January 2025 to update the included studies.

The databases and trial registers that were searched manually included MEDLINE (PubMed), Scopus, Embase (Embase.com), Web of Science, CINAHL (EBSCOhost), and Cochrane Database of Systematic Reviews.

### 3.5 Study selection

All identified citations were collected and uploaded into Rayyan (Qatar Computing Research Institute, Doha, Qatar), where duplicates were removed. The deduplication tool in Rayyan was set at 95 % similarity between articles to consider them duplicates. Following a pilot test, titles and abstracts were screened by two independent reviewers (DP and ABK) for assessment against the inclusion criteria for the review. Potentially relevant sources were retrieved, and their citation details were imported. Two independent reviewers assessed the full text of selected citations in detail. Reasons for excluding sources of evidence in full text were recorded (Appendix 2 – Supplementary Data). Any disagreements between reviewers at each stage of the selection process were resolved through discussion or with the assistance of an additional reviewer (AMA). The results of the search and the study inclusion process were reported in full in the scoping review and presented in a PRISMA-ScR flow diagram.^4^(Fig. 1)

### 3.6 Data collection process

Data was extracted from papers by two independent reviewers, which included specific details about the participants, concept, context, study methods, and key findings relevant to the review questions. The extraction form is provided (see Appendix 3- Supplementary Data).

### 3.7 Data items

A descriptive summary accompanying the tabulated and charted results elucidates their relevance to the review's objectives and research questions. Additionally, an overview of the search results is presented in a table (Table 1, Table 2, Table 3, Table 4, Table 5, Table 6, Table 7), categorized according to the various rehabilitation techniques.

**Table 1:** Summary of the studies.

	STUDY				
Characteristics	Degreef et al^5^	Papapetropoulos et al^6^	Wysocki et al^7^	Shinohara et al^8^	Atzei et al^9^
Type of study	Retrospective	Retrospective	Prospective	Retrospective	Retrospective
Publication year	2005	2010	2012	2013	2015
No of cases	52	27	25	11	48
Mean age	15-56(35.2)	37	30	27	34
Male: Female	24; 27	9; 18	16; 12	7; 4	28; 20
Type of tear	type 1B	type 1B	type 1B	type 1B	type 1B
Mean duration between injury to surgery	6 months	6 months	12 months	10 months	11 months
Type of surgery	Arthroscopic TFCC repair with sutures + K-wire	Arthroscopic TFCC repair with sutures, with 3 PDS sutures	Arthroscopic TFCC repair with sutures, with 3 PDS sutures (outside in)	Arthroscopic TFCC repair with sutures, with 3 PDS sutures (outside in)	Arthroscopic TFCC repair with sutures, with 3 PDS sutures (outside in)
Mean follow-up duration	16 months	24 months	31 months	30 months	33 months
Period and type of complete immobilization	Above elbow cast-3 weeks; below elbow for 3 weeks	Sugar tong splint in 60° of supination −3 weeks	Long arm splint with forearm in supination- 3.5 weeks	Long-arm cast with the elbow at 90°, forearm in neutral rotation, and wrist in neutral-3 weeks	long-arm cast in neutral rotation or slight (45°) supination-1 week
Type of removable splint	Not mentioned	Wrist brace for 3 weeks	Nil	Wrist brace for 8 weeks	Muenster splint-3 weeks
Commencement of elbow ROM	3 weeks	Immediately post-surgery	3.5 weeks	Nil	1 week
Commencement of forearm ROM	6 weeks	3 weeks	3.5weeks	3 weeks	6 weeks
Commencement of wrist ROM	6 weeks	3 weeks	6 weeks	3 weeks	3 weeks
Strengthening exercises	Not mentioned	8 weeks	Not mentioned	Not mentioned	After full recovery of ROM and proprioception
Outcome measures and post-op score (Mean)	DASH<20–71 %,20-40-18 %,>40-15 % Mean VAS-2.43	DASH-9.8 VAS-1.4	DASH-9 VAS-.9	Hand20 score-6 MMWS- excellent = 7(63.6), good = 3(27.2), fair = 1(9 %)	MMWS- excellent = 35 (72.9 %), good = 5(10.4 %), fair = 6 (12.5 %) poor = 2 (4,2 %) vas score −2, DASH score-15
Grip strength	80 % of C/L side	75 % of C/L side	94 % of C/L side	98 % of C/L side	103 % of C/L side
Return to activity	Not mentioned	25 patients at the end of 24 months(92.5 %)	17 patients at the end of 31 months (68 %)	Not mentioned	daily activities by 2months, sports and heavy activities by 3 months
Complications	Not mentioned	paraesthesia in ulnar nerve division = 1	paraesthesia in ulnar nerve division = 2; FCU tendinitis = 1; ECU tendinitis = 1; re-tear due to fall = 1	skin irritation by suture-3	paraesthesia in ulnar nerve division = 5

**Table 2:** Summary of the studies.

	STUDY				
Characteristics	Lee et al^10^	Liu B et al^11^	Tang et al^12^	Yang and Chen^13^	De Araujo et al^14^
Type of study	Retrospective	Retrospective	Retrospective	Retrospective	Retrospective
Publication year	2019	2021	2012	2022	1996
No of cases	46	25	10	12	17
Mean Age	37	28	33.9	32	33
Male: female	29; 17	17; 8	9; 1	7; 5	NA
Type of tear	Type 1B	Type 1B	Type 1B,1D	Type 1B	Type 1 B
Mean duration between injury to surgery	3 months	8 months	8.3 months	5 months	9 months
Type of surgery	Arthroscopic TFCC repair with sutures + PQ advancement	Arthroscopic ligament specific repair	Arthroscopic TFCC repair with double-armed straight needle	Arthroscopic transosseous TFCC repair	Arthroscopic TFCC repair with capsular sutures
Mean follow-up duration	23 months	24 months	8 months	53 months	24 months
Period and type of complete immobilization	Sugar tong splint in 45° of supination – 3 weeks	A sugar-tong splint in semi-supination- 3 weeks	A hinged elbow brace- 3 weeks	Sugar tong −3 weeks	splint not mentioned. Immobilized for 3 weeks
Type of removable splint	Not mentioned	Sugar tong removable splint till 7 weeks	Not mentioned	Not mentioned	Not mentioned
Commencement of elbow ROM	3 weeks	Immediately post-surgery	3 weeks	3 weeks	Not mentioned
Commencement of forearm ROM	3 weeks	4 weeks	3 weeks	3 weeks	3 weeks
Commencement of wrist ROM	3 weeks	4 weeks	3 weeks	3 weeks	3 weeks
Strengthening exercises	After full recovery of ROM and Proprioception	7 weeks	6 weeks	4 weeks	Not mentioned
Outcome measures and post-op score (Mean)	DASH-3 PRWE-11.6	DASH- 7 PRWE - 6 MWS- 95 VAS - 1	MMWS- excellent-2(20 %), good-3(30 %), Fair- 5 (50 %)	DASH -10 MMWS- 95	Not mentioned
Grip strength	92 % OF C/L side	Improvement of 11 kg	Not mentioned	99 % of C/L side	82 % of C/L side
Return to activity	Not mentioned	100 % IN 4–6 MONTHS	100 % returned to employment	Not mentioned	Not mentioned
Complications	Not mentioned	CRPS- 1	paraesthesia in ulnar nerve division-1	Not mentioned	paraesthesia in ulnar nerve division-2

**Table 3:** Summary of the studies.

	STUDY					
Characteristics	Badia and Khanchandani^15^	Estrella et al^16^	Mcadams et al^17^	Iwasaki et al^18^	Yao J and Lee AT^19^	Miwa H et al^20^
Type of study	Prospective	Prospective	Prospective	Retrospective	Retrospective	Retrospective
Publication year	2007	2007	2009	2011	2011	2004
No of cases	23	35	11	12	12	1B-21; 1D-12(33)
Male: Female	14; 9	22; 13	–	6; 6	12; 0	–
Mean age	35	32	23	31	42	33.5
Type of tear	Type-1B	Type-1B,1C,1D	Type 1B,1D	Type 1B	Type-1B	Type-1B,1D
Mean duration between injury to surgery	Not specified	8 months	3 months	24 months	6 weeks	8 months
Type of surgery	Arthroscopic TFCC repair with suture welding	Arthroscopic TFCC repair with sutures	Arthroscopic TFCC repair with sutures-inside out	Arthroscopic TFCC repair with sutures	Arthroscopic TFCC repair with fast fix	Arthroscopic TFCC repair-1B-multiple sutures, 1D-transosseous sutures
Mean follow-up duration	17 months	39months	24 months	30 months	17.5 months	24 months
Period of complete and type of immobilization	Sugar-tong plaster splint in supination with elbow in 90 degrees of flexion-1week, muenster splint-5 weeks	Above elbow cast -neutral to 30 deg supination-3 weeks	Sugar-tong plaster- 2weeks, below elbow cast −4 weeks	Above elbow cast with supination of 45deg-4 weeks	Sugar tong splint for 2 weeks.short arm Munster cast for an 4 weeks	a long arm splint was applied for 3 weeks; short arm splint for another 3 weeks
Type of removable splint	None	Long forearm brace	Not mentioned	2 weeks of removable splint	Not mentioned	Not mentioned
Commencement of elbow ROM	1 week	3 weeks	2 weeks	4 weeks	4 weeks	3 weeks
Commencement of forearm ROM	6 weeks	5–7 weeks	6 weeks	6 weeks	6 weeks	6 weeks
Commencement of wrist ROM	6 weeks	5–7weeks	6 weeks	6 weeks	6 weeks	6 weeks
Strengthening exercises	6 weeks	7 weeks	Not mentioned	Not mentioned	8 weeks	Nil
Outcome measures and post-op score (Mean)	Nil	Vas-3, MWS- excellent-19 (54 %), good −7 (20 %), fair- 4 (11 %), poor −5 (14 %)	mini-DASH scores - 0	MMWS excellent-8(66 %) good-4 (33.3) DASH-7.7	Quickdash score – 11 PRWE-19	Minami criteria- class 1D Excellent-4(33.3 %), good-7(58.3 %), Poor-1(8.3 %) 1B wrists excellent-12(57 %), good-8(38 %), fair-1(4 %)
Grip strength	81 % of C/L side	82 % of C/L side	Not mentioned	103 % of C/L side	64 % of C/L side	88 % of C/L side
Return to activity	100 %	67.8 % patients returned to work at 5 months	100 % in 3 months	58.3 % returned to work at 3.2months	Mean 5 months- (2–9months)	100 %-9.5 weeks
Complications	pain over scar site-1	paraesthesia in ulnar nerve division-6 EDM sutured to dorsal capsule-1	DRUJ instability-1 ECU tendinitis-1	ECU tendinitis-2	Not mentioned	Not mentioned

**Table 4:** Summary of the studies.

	STUDY					
Characteristics	Park JH et al^21^	Dunn et al^22^	Lee et al^10^	Tsai M et al^23^	Yeh et al^24^	Trumble et al^25^
Type of study	Retrospective	Retrospective	Retrospective	Retrospective	Observational	Prospective
Publication year	2018	2019	2019	2021	2022	1997
No of cases	16	15	34	12	201	24
Male: Female	12; 4	13; 2	17; 17	10; 2	156; 47	Not mentioned
Mean age	29.8	21	35	33	26.7	31
Type of tear	Type -1B	TFCC 1B	TFCC 1B	TFCC 1B	TFCC 1B	TFCC 1B,1C,1D
Mean duration between injury to surgery	11 months	44 months	6.8 months	9.8 months	6 WEEKS	4 months
Type of surgery	Arthroscopy assisted transosseous sutures	Arthroscopy assisted transosseous knotless sutures	Arthroscopic suture repair	Arthroscopic repair technique using a pre-tied suture device	Arthroscopic TFCC repair with suture anchor	Arthroscopic TFCC repair with sutures
Mean follow-up duration	31months	3.8 months	27.3 months	15 months	32.6 months	34 months
Period of complete immobilization	Above elbow cast in 30 deg supination-6weeks	Longarm orthosis with the elbow at 90, the forearm in neutral rotation, and the wrist in neutral for 2 weeks, Muenster cast for an additional 4 weeks	Supinated sugar-tong splint at about 45°	Short arm splint - forearm neutral rotation for 6 weeks	Wrist brace with the wrist in a neutral position for 6 weeks	Long-arm cast in 45 deg of supination for 6 weeks
Type of removable splint	Below elbow rigid brace - 2weeks	Not mentioned	Not mentioned	Not mentioned	Not mentioned	Removable wrist brace
Commencement of elbow ROM	6 weeks	Nil	6 weeks	Immediately after surgery	Immediately after surgery	6 weeks
Commencement of forearm ROM	6 weeks	6 weeks	6 weeks	6 weeks	6 weeks	6 weeks
Commencement of wrist ROM	6 weeks	6 weeks	6 weeks	6 weeks	6 weeks	6 weeks
Strengthening exercises	Isometric strengthening exercise at 3 months	Not mentioned	After painfree movements	Not mentioned	.4 weeks post surgery-strengthening and work simulation exercises.	Not mentioned
Outcome measures and post-op score	VAS-.8 MMWS- 83.4 Quick DASH score −9.9	MMWS-84 minidash-9.4 VAS-1.3	DASH-15 PRWE-14	MMWS from 61.3 to 90.4	DASH score- 10.6 MMWS excellent-4(1 %) Good-182 (90.5 %) fair-15 (7 %)	VAS- 1
Grip strength	79 % of C/L side	Not mentioned	91 % of C/L side	Not mentioned	85.4 % of C/L side	Grip strength averaged 85 % C/L side
Return to activity	6 months	93 % returned to work in the military	Not mentioned	91.6 % returned to work	92.5 % returned to work	62.5 % of patients returned to work
Complications	Not mentioned	Re-surgery after trauma-1	Not mentioned	Paraesthesia in ulnar nerve division-2	Not mentioned	Paraesthesia in ulnar nerve division-1, resolved by 4 months

**Table 5:** Summary of the studies.

	STUDY				
Characteristics	Moskal et al^26^	Reiter et al^27^	Short WH^28^	Millants et al^29^	Jegal et al^30^
Type of study	Prospective	Retrospective	Prospective	Retrospective	Retrospective
Publication year	2001	2008	2001	2002	2016
No of cases	20	46	26	35	19
Mean age	33	34	Na	31	37
Male: Female	12; 8	23; 23	Na	15; 20	11; 8
Type of tear	Type 1C	Type 1B	Type 1D	Type 1B	Type 1B
Mean duration between injury to surgery	2.5 years	9.7 months	2 months	6 months	6 months
Type of surgery	Arthrscopic LT ligament repair + k wire fixation	Arthrscopic inside out TFCC repair	Arthroscopic TFCC repair + k wire fixation	Arthroscopic TFCC repair with multiple sutures	Arthroscopic transosseous TFCC repair
Mean follow-up duration	3.1 years	11 months	26 months	58 months	31 months
Period and type of complete immobilization	Sugar-tong splint-3 to 5 days Muenster cast with the forearm in neutral rotation and the wrist in neutral - 4-weeks	Long arm–based wrist splint - 4 weeks	Muenster cast for 8 weeks	Above elbow cast-6 weeks; below elbow cast −3 weeks	Long arm cast with forearm in a neutral rotation or semi-supinated position with the elbow at 90 for 4 weeks
Type of removable splint	Removable Muenster splint for 4 weeks	A Bowers splint- 4 week	A removable volar wrist splint	Not mentioned	Not mentioned
Commencement of elbow ROM	Immediately after surgery	8weeks	Immediately after surgery	6 weeks	4 weeks
Commencement of forearm ROM	8 weeks	8weeks	8 weeks	9 weeks	4 weeks
Commencement of wrist ROM	4 weeks	8weeks	8 weeks	9 weeks	4 weeks
Strengthening exercises	No specific duration	12 weeks	Not mentioned	Nil	Strengthening at 12 weeks
Outcome measures and post-op score (MEAN)	MMWS excellent-13(65 %), good-5(25 %), fair-2(10 %),poor-0	VAS- 3.4 DASH score 21.70	MMWS-excellent-67 %,good-12 %, fair-12 %, poor-8 %	DASH -15 personal wrist score good −29(82.8 %) fair-5(14.2 %) poor −1(2 %)	DASH-11 PRWE - 19, MMWS-excellent −7(36.8 %), good-10(52.6 %) fair −2(10.5 %)
Grip strength	Not mentioned	90 % ofc/L side	90 % ofc/L side	Not mentioned	89 % ofc/L side
Return to activity	Not mentioned	Not mentioned	All returned to work, although 25 % of the patients had some restrictions at work	Not mentioned	Not mentioned
Complications	ECU Tendinitis-4	Paraesthesia in ulnar nerve division-5	Not mentioned	Not mentioned	Not mentioned

**Table 6:** Summary of the studies.

	STUDY				
Characteristics	Jung et al^31^	Shih et al^32^	Abe et al^33^	Yeh CW et al^34^	Soliman et al^35^
Type of study	Retrospective	Not specified	Retrospective	Retrospective	Retrospective
Publication year	2019	2002	2018	2022	2021
No of cases	42	37	29	38	22
Mean Age	35.29	21	30	35	23
Male: Female	29; 13	30; 7	13; 16	33; 5	18; 4
Type of tear	Type 1B	Type-1B,1C,1D	Type-1B	Type 1 B	Type 1B
Mean duration between injury to surgery	6 weeks	10 weeks	7.1 months	8 months	6 weeks
Type of surgery	Arthroscopic transosseous TFCC repair with suture anchor	Arthroscopic transosseous TFCC repair with suture button + k wire fixation	8- open foveal repair; 21-arthroscopic TFCC repair	Arthroscopy-assisted TFCC transcapsular repair with dorsal DRUJ capsule imbrication	Arthroscopic TFCC repair with single strand fibre wire
Mean follow-up duration	26.19 months	25.6 months	34.4 months	35.6 months	29.3 months
Period and type of complete immobilization	Long arm cast with elbow in 90 and forearm in 30 deg supination-4 weeks	Nil	Long-arm cast with 90° of elbow flexion and neutral forearm rotation for 2 weeks. A short arm cast was applied for an additional 2 weeks	A long arm cast was applied with the wrist in neutral position and the elbow in 90 flexion for 4 weeks	Short forearm splint for 4 weeks
Type of removable splint	Nil	Nil	Nil	Nil	Short forearm splint for 2 weeks
Commencement of elbow ROM	4 weeks	Immediately after surgery	2 weeks	5 week	Immediately after surgery
Commencement of forearm ROM	4 weeks	8 weeks	4 weeks	5 week	4 weeks
Commencement of wrist ROM	4 weeks	Immediately after surgery, except for ulnar deviation-started after 2 weeks	4 weeks	5 week	6 weeks
Strengthening exercises	8 weeks	10 weeks	8 weeks	10–12 weeks	8 weeks
Outcome measures and post-op score (Mean)	MMWS- excellent-12, good-18,fair-11, poor-1 scores VAS-1.23 DASH-10.41 PRWE - 9.57	MMWS- excellent-10(27 %) Good-24(65 %) Fair-3(8 %)	NRS was .2 DASH – Open-7.8 Arthroscopy-5.7 MMWS Group O-excellent-100 % Group A 18 excellent-18(85.7 %) good-3(14.2 %)	DASH score 41.6–9.9, PRWE score 37.7–10.3,MMWS 51.3–88.8	VAS score improved from a preoperative score of 7.81 (range 7–9) to a score of 2.78. MMWS improved from preoperative value 56.8 (range 50–70) to a score of 80.5 at final follow-up
Grip strength	83.39 of C/L side	60 %	Group O-96.9 % of C/L side Group A-97.6 % of C/L side	Grip strength 95.1 % of C/L side	69.7 % of C/L side
Return to activity	Not mentioned	Not mentioned	Not mentioned	6 months	100 % at a mean of 29.3 months
Complications	Not mentioned	Broken Kirschner wire that was used to fix the DRUJ-4	Not mentioned	Not mentioned	Not mentioned

**Table 7:** Summary of the studies.

	STUDY			
Characteristics	Bayoumy et al^36^	Lo et al^37^	Shinohara I et al^38^	Yin et al^39^
Type of study	Prospective	Retrospective	Retrospective	Retrospective
Publication year	2016	2021	2024	2024
No of cases	37	99	20	45
Mean age	23.3	35	36	36
Male:Female	29; 8	42; 48	9:11	23:22
Type of tear	Type 1B	Type 1B	Type 1B	Type 1B
Mean duration between injury to surgery	11.1 months	13 months	6 weeks	3.98 months
Type of surgery	Arthroscopic outside-in TFCC repair	Arthroscopic rein-type repair	Arthroscopic transosseous repair with suture tape and swivel lock	Arthroscopic dual-bone tunnel repair
Mean follow-up duration	24 months	13 months	17 months	12 months
Period and type of complete immobilization	A short arm volar splint for 4 weeks	Sugar tong splint for 4 weeks	Sugar-tong orthosis for 3 weeks	Long-arm cast in a neutral position- 3 weeks
Type of removable splint	Plastic brace - 4 weeks	Night-time sugar tong splint	Short wrist splint for 3 weeks	Wrist splint – 3 weeks
Commencement of elbow ROM	Immediately after surgery	4 weeks	Not mentioned	3 weeks
Commencement of forearm ROM	3 months	4 weeks	6 weeks	3 weeks
Commencement of wrist ROM	8 weeks	4weeks	3 weeks	3 weeks
Strengthening exercises	Not mentioned	Strengthening exercises are started after regaining over 70 % ROM	2 months – no mention on the details	3-6 weeks- restricted weight bearing exercises 10 week- weight bearing exercises as tolerated
Outcome measures and post-op score	DASH - 10.2 MMWS -91.2 PRWE – 33 Pain - 2.9	MMWS- 90 quickdash - 0 VAS - 0	MMWS- 91 at 1 year	VAS score 2 DASH - 12 MMWS -90
Grip strength	89 % of C/L side	103 % of C/L side	89 % of C/L side at 1 year	93 % of C/L side at 1 year
Return to activity	Not mentioned	Not mentioned	4.9 months	6 months
Complications	NIL	Superficial infection-1, ulnar nerve neuroma-3, knot irritation-2	Paraesthesia in ulnar nerve division-2 Re-tear-1 after trauma	Paraesthesia in ulnar nerve division-1

**C/L-** Contralateral, DASH- Disabilities of the Arm, Shoulder, and Hand, VAS- Visual Analog Scale, PRWE-Patient rated wrist evaluation, MWS- Mayo Wrist Score, MMWS- Modified Mayo Wrist Score.

### 3.8 Risk of bias in individual studies

Risk of bias assessment was not conducted since scoping reviews focus on providing an overview of available literature rather than synthesizing results.

## 4 Results

Following the search strategy, thirty-five studies were identified between 1996 and 2024 that satisfied the inclusion criteria, of which twenty-five were retrospective,^5^^,^^6^^,^^8^, ^9^, ^10^, ^11^, ^12^, ^13^, ^14^^,^^18^, ^19^, ^20^, ^21^, ^22^, ^23^^,^^27^^,^^29^, ^30^, ^31^^,^^33^, ^34^, ^35^^,^^37^, ^38^, ^39^ eight were prospective,^7^^,^^15^, ^16^, ^17^^,^^25^^,^^26^^,^^28^^,^^36^ one was observational,^24^ and one did not specify the type of study.^32^ The minimum sample size observed was 10^12^, and the maximum was 201.^24^

### 4.1 Pre-operative and per-operative details

One study included children and the adolescent population,^5^ with the youngest being operated at 15 years.^5^ The remaining studies involved an adult population with a mean age of 30 years. The studies reviewed included participants from a diverse range of occupations, except one study that exclusively focused on military personnel as its subjects.^22^

**Fig. 2:**
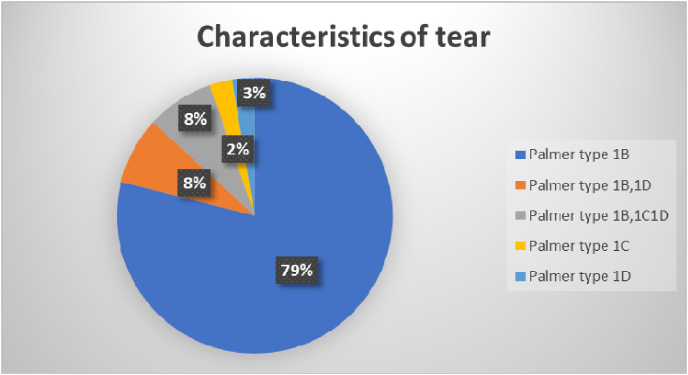
Pie chart depicting the characteristics of tears.

**Fig. 3:**
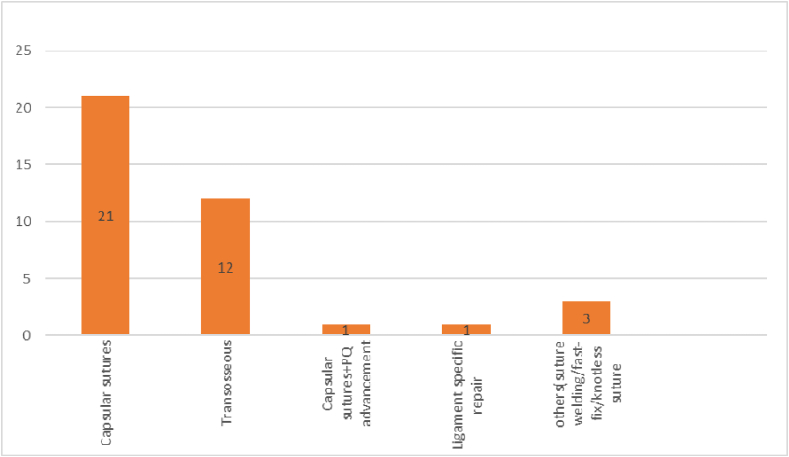
Different types of Arthroscopic TFCC repairs performed in the studies.

The mean duration between the injury and surgery was 8 months, with the surgery being done as early as 6 weeks^22^^,^^24^^,^^31^^,^^35^ and as late as 44 months^22^ months. Twenty-seven papers reported only on Palmar type 1B tear,^5^, ^6^, ^7^, ^8^, ^9^, ^10^, ^11^^,^^13^, ^14^, ^15^^,^^18^^,^^19^^,^^21^, ^22^, ^23^, ^24^^,^^27^^,^^29^, ^30^, ^31^^,^^33^, ^34^, ^35^, ^36^, ^37^, ^38^, ^39^ three each on Palmar type 1B,1D tears,^12^^,^^17^^,^^20^ and Palmar type 1B,1C,1D^16,25,32^and one each on 1C^26^ and 1D^28^ respectively(Fig. 2). The tears were repaired using various arthroscopic-assisted techniques, as illustrated in Fig. 3. The predominant repair methods could be categorized into capsular repairs utilizing non-absorbable sutures or trans-osseous repair techniques. The duration of the final follow-up varied between 8 months^12^ and 58 months.^29^

### 4.2 Rehabilitation

#### 4.2.1 Duration of immobilization

**Fig. 4:**
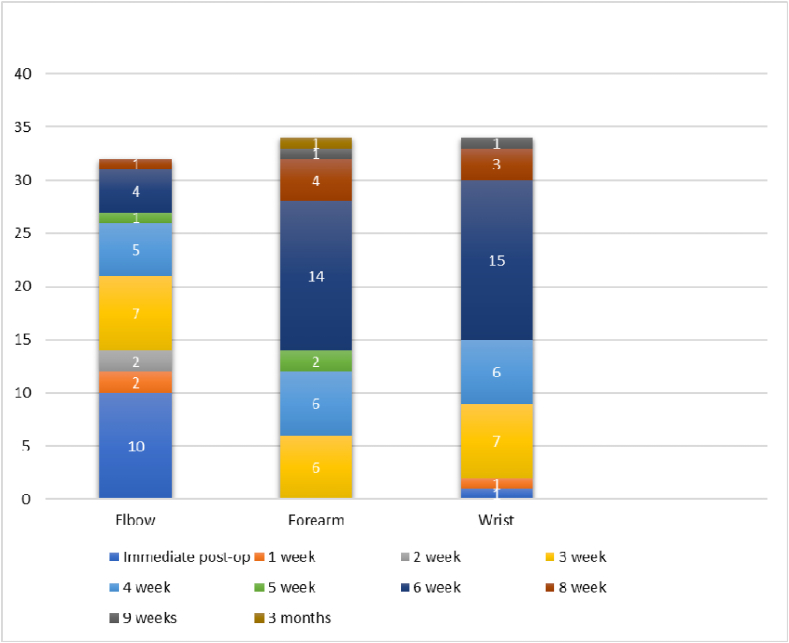
Graphical representation of the various durations of immobilization across the elbow, forearm, and wrist joints.

The duration of immobilization varied across the elbow, forearm, and wrist joints. Most studies, except two, reported on elbow immobilization with differing mobilization initiation. Ten studies initiated the mobilization of the elbow immediately after surgery,^6^^,^^8^^,^^11^^,^^23^^,^^24^^,^^26^^,^^28^^,^^32^^,^^35^^,^^36^ while one other maintained immobilization for as long as eight weeks.^27^ For the forearm, the immobilization timelines varied from 3 weeks^6^ to 3 months.^36^ However, the most frequent period, as reported in fifteen studies, was 6 weeks. Wrist mobilization initiation ranged from immediate initiation following surgery^32^ to a delay of up to eight weeks^36^(Fig. 4).

#### 4.2.2 Splints

**Fig. 5:**
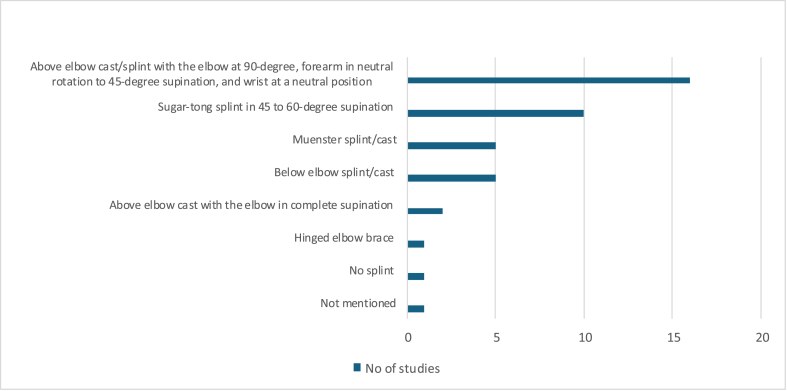
Graphical representation of the different types of splints used across the included studies.

Various types of splints/casts were used for complete immobilization. The above elbow cast or splint, with elbow at 90^0^,forearm in neutral to 45^0^ of supination, and the wrist in a neutral position, was identified as the most commonly immobilized position.^5,8,9,16,18,20–22,25,29–31,33,34,39^The other commonly used splints/casts were a sugar-tong splint in 45^0^ to 60^0^ supination,^6^^,^^10^^,^^11^^,^^13^^,^^15^^,^^17^^,^^19^^,^^26^^,^^37^^,^^38^ below-elbow splint/cast,^23^^,^^24^^,^^27^^,^^35^^,^^36^ Muenster splint/cast^15^^,^^19^^,^^22^^,^^26^^,^^28^ or an above-elbow cast with the forearm in complete supination^7^^,^^15^ respectively. In one study, there was mention of a hinged elbow brace,^12^ another had no splinting,^32^and one other failed to mention the type of immobilization.^14^ Some studies have used a combination of splints/casts, as described in Table 1, Table 2, Table 3, Table 4, Table 5, Table 6, Table 7(Fig. 5).

Subsequently, protected mobilization was initiated along with intermittent use of removable splints, the details of which were provided in fifteen studies only. Short wrist brace^6^^,^^8^^,^^16^^,^^18^^,^^21^^,^^25^^,^^28^^,^^35^^,^^36^^,^^38^^,^^39^ was the most commonly used, followed by either the Muenster,^9^^,^^26^ sugar-tong splints^11^^,^^37^ or the Bower splint^27^ respectively. The period of usage of these splints varied between three weeks to seven weeks, irrespective of the type or the duration of complete immobilization or the type of repair. Nonetheless, the studies lacked specificity regarding the initiation of mobilization, particularly in terms of active, active-assisted, or passive range of motion exercises across the various joints.

#### 4.2.3 Strengthening exercises

A total of twenty-three studies reported on strengthening exercises in rehabilitation protocols. Some of these studies began implementing strengthening exercises only after patients had successfully regained 70–100 % of their wrist and forearm range of motion.^9^^,^^10^^,^^37^ Additionally, the initiation of these exercises was observed to occur up to 12 weeks^21^^,^^27^^,^^30^^,^^34^ at different intervals following surgical procedures, as summarized in Table 1, Table 2, Table 3, Table 4, Table 5, Table 6, Table 7 However, the studies failed to elaborate on the intricate details, such as the group of muscles rehabilitated, the phases of rehabilitation, and the involvement of a hand therapist in the rehabilitation.

### 4.3 Outcome-measures

**Fig. 6:**
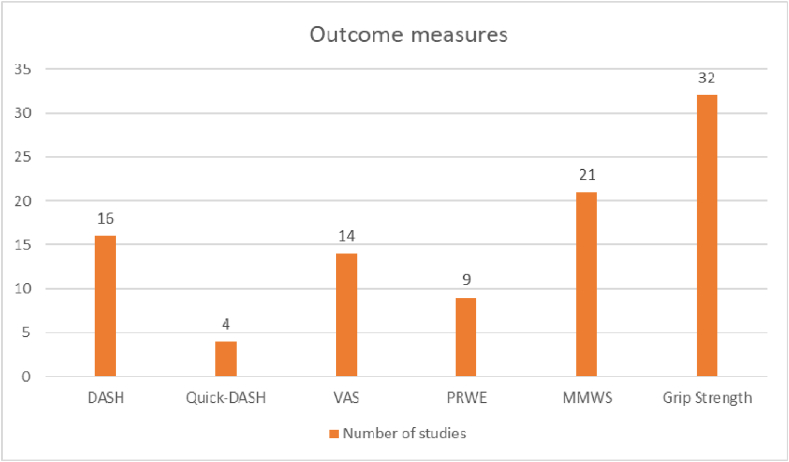
Common outcome measures utilized in the studies.

Various outcome measures have been used across the literature. Patient-reported outcome measures (PROMs) like Disabilities of the Arm, Shoulder, and Hand (DASH), Quick-DASH, Visual Analog Scale (VAS), Hand20 score, Patient Reported Wrist Evaluation (PRWE), and Minami criteria, and physician-reported outcome measures like Mayo Wrist Score (MWS) and Modified Mayo Wrist Score(MMWS) were used for reporting(Fig. 6).

A total of sixteen studies reported outcomes utilizing the DASH score, yielding a mean score of 10.9, as documented at the time of final follow-up.^5^, ^6^, ^7^^,^^9^, ^10^, ^11^^,^^13^^,^^18^^,^^24^^,^^27^^,^^29^, ^30^, ^31^^,^^34^^,^^36^^,^^39^ Quick-DASH was used to report outcomes in four studies, with an average score of 8.14^19,21,22,37^.

The VAS score was documented across fourteen studies, yielding a mean score of 1.66 at the final follow-up.^5^, ^6^, ^7^^,^^9^^,^^11^^,^^16^^,^^21^^,^^22^^,^^25^^,^^27^^,^^31^^,^^35^^,^^38^^,^^39^

A total of eight studies reported final outcomes using the PRWE score, yielding an average score of 10.9^10^, ^11^, ^19^, ^30^, ^31^, ^34^, ^36^. One study reported using Hand20 score^8^ and another one used Minami criteria.^20^

The MMWS score was utilized to report physician-based outcome measures in a total of twenty-one studies. Among these studies, 44 % of participants achieved excellent outcome scores, while 42 % attained good scores. Additionally, eight studies presented only the final MMWS scores, which yielded an average of 87.9^9^^,^^12^^,^^13^^,^^13^, ^21^, ^22^, ^23^, ^24^^,^^26^^,^^28^^,^.^30^, ^31^, ^32^, ^33^, ^34^, ^35^, ^36^, ^37^, ^38^ The MWS score was used to report outcomes in two studies.^11^^,^^16^

Grip strength was the most consistently reported objective outcome measure. The recovery in the grip strength of patients was an average of 85 % of the contralateral side (60–103 %).

### 4.4 Return to activity

Twenty-one studies reported on the return to work. An average of 87 % of the operated population returned to work. However, the duration of return-to-work varied from 3 months^9^ to 31 months.^7^ A study conducted by Dunn et al.,^22^ focused exclusively on military personnel and revealed that 93 % of the participants successfully returned to their work within 44 months.

### 4.5 Complications

**Fig. 7:**
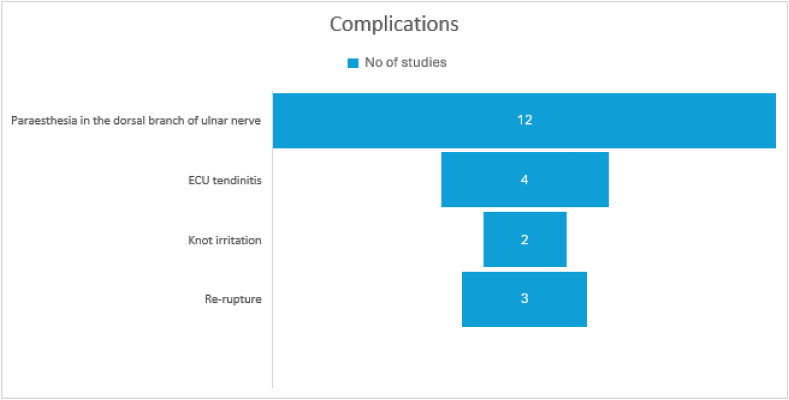
Graph depicting the common complications noted in the studies.

Twenty studies reported on the complications, of which paraesthesia in the dorsal branch of the ulnar nerve division(DBUN) was reported to be the most common.^6^^,^^7^^,^^9^^,^^12^^,^^14^^,^^16^^,^^23^^,^^25^^,^^27^^,^^37^, ^38^, ^39^This was followed by ECU tendinitis^7^^,^^16^^,^^17^^,^^26^ and knot irritation.^8^^,^^37^ However, these complications were transient. One case of re-rupture each was reported in three studies following re-injury.^7^^,^^22^^,^^39^ (Fig. 7).

## 5 Discussion

With the evolution of the anatomical knowledge of TFCC and advancing techniques for its repair, promising improvement has been observed in the outcomes of the surgery. However, there is still ambiguity in various aspects related to TFCC repair and rehabilitation.

This scoping review provides insight into the current practices on the management of TFCC repair and highlights the heterogeneity in the surgical repair techniques, the post-operative regimes of immobilization, rehabilitation, and outcome measures. Considering the remarkable progress in recent times on this commonly encountered clinical condition, the report underscores the need for further evidence-based, strong scientific methods to improve the quality of care in patients with TFCC tears.

Contrary to the belief that only peripheral tears in the outer 20 % can be repaired(Palmer 1B),^40^ we noted studies where Palmer types 1C and 1D are also amenable to repair,^12^^,^^16^^,^^20^^,^^25^^,^^26^^,^^28^^,^^32^ though Palmer type 1B is the most common TFCC tear to be repaired. In addressing ulnar-sided tears, a range of operative techniques are available(i.e., capsular repair, transosseous repair) and achievable through arthroscopic techniques. The outcomes of these approaches have been shown to be comparably effective, allowing for flexibility in surgical planning based on patient-specific factors and the surgeon's expertise. However, radial-sided tears are repaired only by trans-osseous sutures through the radial side.

McCarron et al.,^41^in their scoping review concluded that most of the studies initiated wrist and forearm ROM at an average of 6 weeks, with 80 % commencing during the proliferation or maturation phase of the ligament healing process. In our study, we observed that the immobilization period could be classified into period of complete immobilization and restricted mobilization, where removable splints were used along with the initiation of mobilization. A consistent period of immobilization was observed across all studies, regardless of age, tear type, or repair method. However, the duration varied among the elbow, forearm, and wrist joints, even for similar tears and repairs, as summarized in Table 1, Table 2, Table 3, Table 4, Table 5, Table 6, Table 7 This variability underscores the importance of a nuanced understanding of joint dynamics and the ligament healing process to determine a common period of immobilization.

While the timeline for initiating rehabilitation exercises varied widely, none of the studies offered information on the type of mobilization used (active, active-assisted, or passive), the duration of strengthening exercises, the specific exercises performed, or the targeted muscles during this rehabilitation phase. This omission may be attributed to the limitations of the included study's scope or the fact that a hand therapist primarily addressed these details. The distal radio-ulnar joint is deemed stable in a mid-prone to supinated position of the forearm. Following TFCC repair, it is recommended to avoid the extremes of protonation or supination to minimize stress on the healing ligaments. Therefore, immobilization in a semi-supinated position or at 45 degrees of supination is considered appropriate.^42^ A review of the studies included shows that the commonly used immobilization position has the elbow at a 90-degree flexion, with the forearm placed in neutral to 45 degrees of supination. Nonetheless, there remains a lack of consensus among the studies regarding the optimal positioning of the limb during immobilization, in addition to the selection and duration of removable splints.

The quality and parameters used in outcome measures have improved over time, reflecting the consideration of not only the recovery of the impairment of the affected limb but also the improvement in the socio-cultural environment of the patient.Earlier studies relied on non-specific wrist scores and PROMs such as VAS, DASH, and quick-DASH. However, more recent studies have shifted towards additional use of wrist-specific scores like the PRWE score and the MMWE, as outlined in Table 1, Table 2, Table 3, Table 4, Table 5, Table 6, Table 7 However, grip strength is one outcome measure that has been reported consistently across most of the studies. Considering these variations, there is a need for more consistent use of validated and rigorously tested outcome measures and incorporating them in guidelines for the assessment of such injuries for future studies.

At the final follow-up, all studies showed favorable post-operative outcome scores. However, most were retrospective, making it difficult to quantify improvement due to missing pre-operative scores. Besides, substantial variability in the duration of follow-up periods complicated the efforts to identify a definitive threshold at which the improvement in outcome scores plateaued. We noted a cumulative result of 87 % of patients returning to pre-injury level. However, the duration of return to work varied between the studies, ranging from 3 months to 31 months.

Most of the complications noted were associated with the surgery, caused by injury or inflammation of the DBUN or by irritation from the prominence of the suture used during the inside-out technique. However, all of them resolved on their own at an average duration of six months. This information suggests the need to refine the techniques used in arthroscopy to protect the DBUN. It is heartening to note that the rate of re-rupture is very low after repair, underlying the gratifying results of standard repair techniques.

This review has several limitations. Since the outcome measures vary across the studies, it is not possible to draw a definitive conclusion regarding the most effective type of surgery. Therefore, level 1 or 2 studies are needed to provide further justification. Additionally, this review does not include studies involving malunion of the distal end radius and/or ulna, non-union of the ulnar styloid, or cases of TFCC repair alongside bony procedures such as ulnar shortening osteotomy. As a result, many primary studies are excluded from this review, necessitating further research to assess the outcomes of TFCC repairs in these specific situations.

The scoping review emphasizes the variability present in various aspects of rehabilitation following TFCC repair. Therefore, conducting a multicentric study to standardize the rehabilitation protocol for a similar type of repair (Eg- Capsular repair, trans-osseous repair) is recommended. Additionally, a Randomized Controlled Trial to evaluate the outcomes of mobilization at different stages of ligament healing would be ideal, with a focus on determining the minimum duration of immobilization necessary to promote optimal recovery and effectively restore function. Furthermore, prospective studies could investigate the time of return to work associated with different types of repairs, such as capsular repair of the TFCC versus trans-osseous repair.

## 6 Conclusion

Through this scoping review, we identified that Palmar types 1B, 1C, and 1D are suitable for repair using various arthroscopy-assisted procedures. The duration of immobilization and rehabilitation varied among studies, even for similar repairs, and often overlooked the biological aspects of ligament healing. However, the most common immobilization period was 6 weeks. Commonly utilized outcome measures included the DASH, MMWS, and grip strength assessments. Notably, paraesthesia in the dorsal branch of the ulnar nerve (DBUN) emerged as the most frequently reported complication. Additionally, 87 % of the patient population who underwent surgery successfully returned to work, with an average recovery period of 9.6 months.

## Declaration of patient consent form

Not applicable.

## Informed consent

Not applicable.

## Guardian/Parent's consent

This is not applicable to this study.

## Credit author statement contributorship

Dr. Deepthi Paraj(DP): wrote the first draft of the manuscript. All authors reviewed and edited the manuscript and approved the final version of the manuscript, Protocol development, addressing the conflicts as a third reviewer during the screening process, Dr. Anil K Bhat(AKB)- researched literature and conceived the study. Screening of the studies individually.Protocol development, addressing the conflicts as a third reviewer during the screening process, Dr. Ashwath M Acharya(AMA),Protocol development, addressing the conflicts as a third reviewer during the screening process.researched literature and conceived the study. Screening of the studies individually.

## Guarantor

Dr. Anil K Bhat.

## Ethical approval

Not applicable.

## Ethical approval

Not applicable for this study.

## Funding

This research did not receive any specific grant from funding agencies in the public, commercial, or not-for-profit sectors.

## Declaration of competing interest

The authors declare no potential conflicts of interest with respect to the research, authorship, and/or publication of this article.
