# Supplementary material for: Clinical and functional outcomes of rehabilitation strategies following arthroscopic repair of chronic isolated peripheral TFCC tears: A scoping review
Source: J Orthop. 2025 Jul 5;66:310–22. doi: 10.1016/j.jor.2025.06.028 (PMC12274733; doi:10.1016/j.jor.2025.06.028)
Supplement: Multimedia component 1 [file mmc1.docx]

**Appendix I: Search strategy**

**Pubmed/MEDLINE (Ovid)#**

**Date- 22^nd^ Feb 2024**

| **Search** | **Query** | **Records retrieved** |
| --- | --- | --- |
| #1 | ("TFCC"[All Fields]) OR ("triangular"[All Fields] OR "triangularity"[All Fields]) AND "fibro*"[All Fields]) OR (("triangular fibrocartilage"[MeSH Terms] OR ("triangular"[All Fields] AND "fibrocartilage"[All Fields]) OR "triangular fibrocartilage"[All Fields] OR ("triangular"[All Fields] AND "fibrocartilage"[All Fields] AND "complex"[All Fields]) OR "triangular fibrocartilage complex"[All Fields]) | 1,884 |
| #2 | (("TFCC"[All Fields] OR (("triangular"[All Fields] OR "triangularity"[All Fields]) AND "fibro*"[All Fields] OR (("triangular fibrocartilage"[MeSH Terms] OR ("triangular"[All Fields] AND "fibrocartilage"[All Fields]) OR "triangular fibrocartilage"[All Fields] OR ("triangular"[All Fields] AND "fibrocartilage"[All Fields] AND "complex"[All Fields]) OR "triangular fibrocartilage complex"[All Fields]) AND ("repairability"[All Fields] OR "repairable"[All Fields] OR "repaire"[All Fields] OR "repaired"[All Fields] OR "repairment"[All Fields] OR "wound healing"[MeSH Terms] OR ("wound"[All Fields] AND "healing"[All Fields]) OR "wound healing"[All Fields] OR "repair"[All Fields] OR "repairing"[All Fields] OR "repairs"[All Fields])) | 397 |
| #3 | ("exercise"[MeSH Terms] OR "exercise"[All Fields] OR "exercises"[All Fields] OR "exercise therapy"[MeSH Terms] OR ("exercise"[All Fields] AND "therapy"[All Fields]) OR "exercise therapy"[All Fields] OR "exercising"[All Fields] OR "exercise s"[All Fields] OR "exercised"[All Fields] OR "exerciser"[All Fields] OR "exercisers"[All Fields] OR ("rehabilitant"[All Fields] OR "rehabilitants"[All Fields] OR "rehabilitate"[All Fields] OR "rehabilitated"[All Fields] OR "rehabilitates"[All Fields] OR "rehabilitating"[All Fields] OR "rehabilitation"[MeSH Terms] OR "rehabilitation"[All Fields] OR "rehabilitations"[All Fields] OR "rehabilitative"[All Fields] OR "rehabilitation"[MeSH Subheading] OR "rehabilitation s"[All Fields] OR "rehabilitational"[All Fields] OR "rehabilitator"[All Fields] OR "rehabilitators"[All Fields]) OR ("mobilisation"[All Fields] OR "mobilisations"[All Fields] OR "mobilise"[All Fields] OR "mobilised"[All Fields] OR "mobiliser"[All Fields] OR "mobilisers"[All Fields] OR "mobilises"[All Fields] OR "mobilising"[All Fields] OR "mobilization"[All Fields] OR "mobilizations"[All Fields] OR "mobilize"[All Fields] OR "mobilized"[All Fields] OR "mobilizer"[All Fields] OR "mobilizers"[All Fields] OR "mobilizes"[All Fields] OR "mobilizing"[All Fields]) OR ("immobile"[All Fields] OR "immobilisation"[All Fields] OR "immobilization"[MeSH Terms] OR "immobilization"[All Fields] OR "immobilise"[All Fields] OR "immobilised"[All Fields] OR "immobiliser"[All Fields] OR "immobilises"[All Fields] OR "immobilising"[All Fields] OR "immobilisations"[All Fields] OR "immobilize"[All Fields] OR "immobilizations"[All Fields] OR "immobilized"[All Fields] OR "immobilizer"[All Fields] OR "immobilizers"[All Fields] OR "immobilizes"[All Fields] OR "immobilizing"[All Fields]) OR ("therapeutics"[MeSH Terms] OR "therapeutics"[All Fields] OR "therapies"[All Fields] OR "therapy"[MeSH Subheading] OR "therapy"[All Fields] OR "therapy s"[All Fields] OR "therapys"[All Fields])) AND (1981:2024/2/22[pdat]) | 10,754,490 |
| #4 | #2 and #3 | 310 |
| Limited to #date, language limits etc.# | |  |

**Cochrane**

**Date- 22^nd^ Feb 2024**

| **Search** | **Query** | **Records retrieved** |
| --- | --- | --- |
| #1 | TFCC OR TRIANGULAR FIBROCARTILAGE COMPLEX OR TRIANGULAR FIBRO* | 51 |
| #2 | TRIANGULAR FIBRO* | 75 |
| #3 | REPAIR | 23380 |
| #4 | EXERCISE OR REHABILITATION OR MOBILIZATION OR IMMOBILISATION OR THERAPY | 1054073 |
| #5 | #1 OR #2 AND #3 AND #4 | 52 |
| Limited to #date, language limits etc.# | |  |

**Embase**

**Date- 22^nd^ Feb 2024**

| **Search** | **Query** | **Records retrieved** | |  |
| --- | --- | --- | --- | --- |
| #1 | ((tfcc OR triangular) AND fibro* OR triangular) AND fibrocartilage AND complex))) | 1,350 | |  |
| #2 | Repair | 605,830 | |  |
| #3 | 'exercise'/exp OR exercise OR 'rehabilitation'/exp OR rehabilitation OR 'mobilization'/exp OR mobilization OR 'immobilisation'/exp OR immobilisation OR 'therapy'/exp OR therapy) | 13,910,914 | |  |
| #4 | #1 AND #2 AND #3 | 136 | |  |
| Limited to #date, language limits etc.# | | |  | |

**Scopus**

**Date- 23^nd^ Feb 2024**

| **Search** | **Query** | **Records retrieved** |
| --- | --- | --- |
| #1 | ( ALL ( tfcc OR triangular AND fibro* OR triangular AND fibrocartilage AND complex ) | 3555 |
| #2 | Repair | 715,421 |
| #3 | (exercise OR rehabilitation OR mobilization OR immobilisation OR therapy ) | 7,100,986 |
| #4 | ( ( ALL ( tfcc OR triangular AND fibro* OR triangular AND fibrocartilage AND complex ) AND TITLE-ABS-KEY ( repair ) )  AND ( TITLE-ABS-KEY ( exercise OR rehabilitation OR mobilization OR immobilisation OR therapy ) ) | 78 |
| Limited to #date, language limits etc.# | |  |

**Web of science**

**Date- 23^nd^ Feb 2024**

| **Search** | **Query** | **Records retrieved** |
| --- | --- | --- |
| #1 | ALL=(TFCC OR TRIANGULAR FIBRO* OR TRIANGULAR FIBROCARTILAGE COMPLEX) | 2,243 |
| #2 | ALL=(repair) | 640,439 |
| #3 | ALL=(EXERCISE OR REHABILITATION OR MOBILIZATION OR IMMOBILISATION OR THERAPY) | 4,751,079 |
| #4 | #1 AND #2 AND #3 | 146 |
| Limited to #date, language limits etc.# | |  |

**CINAHL Ultimate (EBSCOhost Research Databases)**

**Date- 23^nd^ Feb 2024**

| **Search** | **Query** | **Records retrieved** |
| --- | --- | --- |
| #1 | TFCC OR TRIANGULAR FIBRO* OR TRIANGULAR FIBROCARTILAGE COMPLEX | 373 |
| #2 | Repair | 61,001) |
| #3 | EXERCISE OR REHABILITATION OR MOBILIZATION OR IMMOBILISATION OR THERAPY | 2,046,141 |
| #4 | #1 AND #2 AND #3 | 21 |
| Limited to #date, language limits etc.# | |  |

**Additional records identified through other sources**

| Journal | Search term | Search result | Additional records |
| --- | --- | --- | --- |
| Journal of Hand Surgery | Triangular fibrocartilage complex or TFCC and Repair | 151 | 10 |
| Journal of Hand Surgery Global Online | Triangular fibrocartilage complex or TFCC and Repair | 14 | 2 |
| Journal of Hand Surgery: European | Triangular fibrocartilage complex or TFCC and Repair | 167 | 8 |
| Techniques in Hand & Upper Extremity Surgery | Triangular fibrocartilage complex or TFCC and Repair | 41 | 2 |
| The Journal of Arthroscopic & Related Surgery | Triangular fibrocartilage complex or TFCC and Repair | 90 | 0 |
| Hand Clinics | Triangular fibrocartilage complex or TFCC and Repair | 149 | 0 |
| Journal of Hand therapy | Triangular fibrocartilage complex or TFCC and Repair | 28 | 0 |
| HAND | Triangular fibrocartilage complex or TFCC and Repair | 77 | 1 |
| INJURY | Triangular fibrocartilage complex or TFCC and Repair | 18 | 0 |
| Total | | | 23 |

**Appendix II: Studies ineligible following full-text review**

1. Zhu JQ, Ma ZH, Xing LF, Liu YH, Wang XL, Dai SY, et al. [Arthroscopic treatment for post-traumatic chronic wrist pain]. Zhongguo Gu Shang. 2011;24(9):726–8 *– Includes all wrist conditions.*
2. Zhao J, Lin Y, Li L, Huang Y. A new arthroscopic repair technique for triangular fibrocartilage complex using an intracapsular suture: an outside-in transfer all-inside repair. Journal of orthopaedic surgery and research. 2023;18(1):896.-*Surgical note*
3. Zhang W, Hou P, Wang C, Wu T, Ho PC, Sun L. Arthroscopic one-tunnel transosseous reconstruction of chronic triangular fibrocartilage complex foveal tears: outcomes in 12 patients. J Hand Surg Eur Vol. 2022;47(3):296–301.- *TFCC reconstruction and not repair.*
4. Yao J, Dantuluri P, Osterman A, Yao J, Dantuluri P, Osterman AL. A novel technique of all-inside arthroscopic triangular fibrocartilage complex repair. ARTHROSCOPY-THE JOURNAL OF ARTHROSCOPIC AND RELATED SURGERY. 2007;23(12). *Surgical note*
5. Xu T, Pan X, Mi J, Xu T, Pan X, Mi J. Improved Outside-In Suture through the Joint Capsule to Repair the Palmer I-B Triangular Fibrocartilage Complex Superficial Injury. COMPUTATIONAL AND MATHEMATICAL METHODS IN MEDICINE. 2021;2021. – *paper retracted*
6. Wu M, Miller PE, Waters PM, Bae DS. Early Results of Surgical Treatment of Triangular Fibrocartilage Complex Tears in Children and Adolescents. J Hand Surg Am. 2020;45(5):449.e1-449.e9. – *associated bony procedures*
7. Whipple TL, Geissler WB. Arthroscopic management of wrist triangular fibrocartilage complex injuries in the athlete. Orthopedics. 1993;16(9):1061–7.- *Not available*
8. Wedemeyer A, Wedemeyer C, Heckelei W, Preissler P. [Arthroscopic refixation of traumatic 1B-lesions of the TFCC--a retrospective study]. Handchir Mikrochir Plast Chir. 2009;41(3):135–40.- *not available in English*
9. Unglaub JM, Bruckner T, Heyse TJ, Eysel P, Langer MF, Spies CK. Long-term results of more than 13 years after arthroscopic repair of triangular fibrocartilage complex (TFCC) Palmer 1B tears: a comparison with short- and mid-term results. Eur J Trauma Emerg Surg. 2022;48(3):2309–17*.- long term follow up of previous study*
10. Trumble T. Radial side (1D) tears. Hand Clin. 2011;27(3):243–54.- *book chapter*
11. Theumann N, Kamel EM, Bollmann C, Sturzenegger M, Becce F. Bucket-handle tear of the triangular fibrocartilage complex: Case report of a complex peripheral injury with separation of the distal radioulnar ligaments from the articular disc. Skelet Radiol. 2011;40(12):1617–21.- *Single case, no follow up*
12. Terry CL, Waters PM. Triangular fibrocartilage injuries in pediatric and adolescent patients. J Hand Surg Am. 1998;23(4):626–34.- *associated with fractures*
13. Tawonsawatruk T, Phoophiboon P, Kanchanathepsak T, Tuntiyatorn P, Tawonsawatruk T, Phoophiboon P, et al. Comparative Analysis of Treatment Outcomes: Modified Ulnar Gutter Slab vs. Sugar Tong Slab for Distal Radioulnar Joint Instability Following Triangular Fibrocartilage Complex Repair. JOURNAL OF CLINICAL MEDICINE. 2023;12(20). *– No surgical procedure*
14. Tang CYK, Fung B, Rebecca C, Lung CP. Another light in the dark: Review of a new method for the arthroscopic repair of triangular fibrocartilage complex. J Hand Surg (USA). 2012;37(6):1263–8. -*Review article*
15. Srinivasan R, Shrouder-Henry J, Richard M, Ruch D, Srinivasan RC, Shrouder-Henry JJ, et al. Open and Arthroscopic Triangular Fibrocartilage Complex (TFCC) Repair. JOURNAL OF THE AMERICAN ACADEMY OF ORTHOPAEDIC SURGEONS. 2021;29(12):518–25.- *Review article*
16. Soreide E, Husby T, Haugstvedt JR. A long-term (20 years’) follow-up after arthroscopically assisted repair of the TFCC. Journal of Plastic Surgery and Hand Surgery. 2017;51(5):296–300. – *follow up of previous study*
17. Shojaie B, Bazzi N, Saremi H. A Simple Surgical Technique for Arthroscopic Repair of Traumatic Radial-Side Peripheral TFCC Tear. Tech Hand Up Extrem Surg. 2023;27(4):200–3. – *Surgical note*
18. Schmelzer-Schmied N, Schmelzer-Schmied N. Arthroscopic refixation of TFCC by bone screw anchor. OPERATIVE ORTHOPADIE UND TRAUMATOLOGIE. 2016;28(4):251–62. – *not available in English*
19. Ryoo HJ, Kim YB, Kwak D, Choi IC, Park JW. Ulnar positive variance associated with TFCC foveal tear. Skeletal Radiol. 2023;52(8):1485–91.-*Radiological study*
20. Ruch DS, Papadonikolakis A. Arthroscopically assisted repair of peripheral triangular fibrocartilage complex tears: factors affecting outcome. Arthroscopy. 2005;21(9):1126–30. *– no proper rehabilitation*
21. Roh YH, Yun YH, Kim DJ, Nam M, Gong HS, Baek GH. Prognostic factors for the outcome of arthroscopic capsular repair of peripheral triangular fibrocartilage complex tears. Archives of Orthopaedic and Trauma Surgery. 2018;138(12):1741–6- *retracted*
22. Pillukat T, Fuhrmann R, Windolf J, van Schoonhoven J, Pillukat T, Fuhrmann RA, et al. Arthroscopically assisted transcapsular refixation of the triangular fibrocartilage complex of the wrist. OPERATIVE ORTHOPADIE UND TRAUMATOLOGIE. 2016;28(4):233–50. – *not in English*
23. Peterson R, Savoie F, Field L, Peterson R, Savoie F, Field L. Arthroscopic treatment of sports injuries to the triangular fibrocartilage. SPORTS MEDICINE AND ARTHROSCOPY REVIEW. 1998;6(4):262–9. – *not a primary study*
24. Pederzini LA, Tosi M, Prandini M, Botticella C. All-inside suture technique for Palmer class 1B triangular fibrocartilage repair. Arthroscopy. 2007;23(10):1130.e1-4. -surgical note
25. Papapetropoulos PA, Ruch DS. Repair of arthroscopic triangular fibrocartilage complex tears in athletes. Hand Clin. 2009;25(3):389–94.- *Book Chapter*
26. Paley D, Rubenstein J, McMurtry RY. Irreducible dislocation of distal radial ulnar joint. ORTHOP REV. 1986;15(4):228–31-*no TFCC tears*.
27. Pajares S, Martínez-Catalán N, Novo-Rivas U, Pajares S, Martinez-Catalan N, Novo-Rivas U. Stabilization for acute distal radioulnar instability: A novel surgical technique *. INJURY-INTERNATIONAL JOURNAL OF THE CARE OF THE INJURED. 2021;52:S137–44. -*Associated fractures*
28. Nam JJ, Choi IC, Kim YB, Park JW. Clinical Outcomes of Arthroscopic One-Tunnel Triangular Fibrocartilage Complex Transosseous Suture Repair Are Not Diminished in Cases of Ulnar Styloid Process Fracture Nonunion. Arthroscopy - Journal of Arthroscopic and Related Surgery. 2023;39(1):32–8.-*No immobilization, no rehab protocol*
29. Nakamura T, Sato K, Okazaki M, Toyama Y, Ikegami H. Repair of foveal detachment of the triangular fibrocartilage complex: Open and arthroscopic transosseous techniques. Hand Clinics. 2011;27(3):281–90- *Book Chapter.*
30. Nakamura T, Nakao Y, Ikegami H, Sato K, Takayama S. Open repair of the ulnar disruption of the triangular fibrocartilage complex with double three-dimensional mattress suturing technique. Tech Hand Upper Extremity Surg. 2004;8(2):116–23.- *Technique of repair*
31. Nagle D, Nagle D. Triangular fibrocartilage complex tears in the athlete. CLINICS IN SPORTS MEDICINE. 2001;20(1):155-+. *-not a primary study*
32. Moritomo H. Advantages of open repair of a foveal tear of the triangular fibrocartilage complex via a palmar surgical approach. Tech Hand Up Extrem Surg. 2009;13(4):176–81. -*only surgical note*
33. Monsivais JJ, Herber A, Charest G, Ogunleye D, Weaver M. Comparative Study of 2 Bone Anchors Using a Limited Open Procedure for the Management of Distal Radioulnar Joint Instability. Hand (N Y). 2022;17(1):75S-80S.-*No details on splints*
34. 55. Moloney M, Farnebo S, Adolfsson L. 20-Year outcome of TFCC repairs. J Plast Surg Hand Surg. 2018;52(3):193–7.
35. Möldner M, Unglaub F, Hahn P, Müller LP, Bruckner T, Spies CK. Functionality after arthroscopic debridement of central triangular fibrocartilage tears with central perforations. J Hand Surg (USA). 2015;40(2):252-258.e2. – *un-repairable tears*
36. Millants P, De Smet L, Van Ransbeeck H. Outcome study of arthroscopic suturing of ulnar avulsions of the triangular fibrocartilage complex of the wrist. Chir Main. 2002;21(5):298–300. – *no clinical follow up*
37. McCarron L, Coombes BK, Bindra R, Bisset L. Current rehabilitation recommendations following primary triangular fibrocartilage complex foveal repair surgery: A survey of Australian hand therapists. Journal of Hand Therapy. 2023;36(4):932–9. - *questionnaire*
38. Marès O, Bosch C. Distal radioulnar joint instability: Diagnosis and treatment of acute and chronic lesions. Orthopaedics and Traumatology: Surgery and Research [Internet]. 2023;109(1).- *No rehabilitation protocol*
39. Mahmoud M, Ezzat M, Elnasr AS, Abdel-Wahed M, Rizk A, Mohsen I. Trans - 6R Portal Repair of Superficial TFCC Tears; A Modified Arthroscopic Outside-in Technique. Arthroscopy Techniques. 2022;11(12):e2225–32. *– Technical note*
40. Luchetti R, Atzei A, Cozzolino R, Fairplay T, Badur N, Luchetti R, et al. Comparison between open and arthroscopic-assisted foveal triangular fibrocartilage complex repair for post-traumatic distal radio-ulnar joint instability. JOURNAL OF HAND SURGERY-EUROPEAN VOLUME. 2014;39(8):845–55.- *Associated fractures and ulnar styloid non-union.*
41. Lu C, Zhang H, Zhang L, Wang P, Wang X. [Anatomical repair of Atzei-EWAS type 2 triangular fibrocartilage complex injury under wrist arthroscopy]. Zhongguo Xiu Fu Chong Jian Wai Ke Za Zhi. 2021;35(11):1417–21. – *not English*
42. Lee KH, Shim BJ, Gong HS. Open Foveal Repair of the Triangular Fibrocartilage Complex Tears Associated with Symptomatic Ulnar Styloid Non-union. J Hand Surg Asian Pac Vol. 2022;27(2):248–55. – *associated bony injuries*
43. Kwon BC, Lee JH, Lee SY. What Is the Effect of the Ulnar-Plus Variance on the Outcomes of Arthroscopic Repair of the Peripheral Ulnar-Side Triangular Fibrocartilage Complex Tear? Arthroscopy. 2020;36(9):2415–22.- *out of scope of study*
44. Kovachevich R, Elhassan BT. Arthroscopic and open repair of the TFCC. Hand Clinics. 2010;26(4):485–94.—*book chapter*
45. Ko J, Wiedrich T, Ko JH, Wiedrich TA. Triangular Fibrocartilage Complex Injuries in the Elite Athlete. HAND CLINICS. 2012;28(3):307- *technical note*
46. Khair Y, Mustafa A, Mestrihi S, Azzam E, Al-Qasaimeh M, Awad D, et al. Outcome in TFCC repair using micro anchor and trans-osseous technique. EXPERIMENTAL AND THERAPEUTIC MEDICINE. 2023;26(6). *– no rehabilitation*
47. KCT0003241. Factors affecting surgical outcomes of TFCC injury. http://www.who.int/trialsearch/Trial2.aspx?TrialID=KCT0003241 [Internet]. 2018; -*associated bony procedures*
48. Kaempf R, Atzei A, Brunelli J, Delgado P, Kaempf R, Atzei A, et al. Arthroscopic Treatment of Traumatic Isolated Volar Dislocation of the Distal Radioulnar Joint: Case Report and Management Proposal. JOURNAL OF WRIST SURGERY. 2024 *- Single case report*
49. Kabakas F, Özçelik I, Ugurlar M, Mersa B, Yazar M, Uzun M, et al. Results of arthroscopic repair of triangular fibrocartilage complex peripheral tears (Palmer type 1B). ULUSAL TRAVMA VE ACIL CERRAHI DERGISI-TURKISH JOURNAL OF TRAUMA & EMERGENCY SURGERY. 2014;20(3):205–10.- *Not in English*
50. Jung H, Park J, Park H, Lee J, Jung HS, Park JG, et al. Postoperative immobilization using a short-arm cast in the semisupination position is appropriate after arthroscopic triangular fibrocartilage complex foveal repair. BONE & JOINT JOURNAL. 2022;104(2):249–56. – *Different mobilizations in one study*
51. Jantea CL, Baltzer A, Rüther W. Arthroscopic repair of radial-sided lesions of the triangular fibrocartilage complex. Hand Clin. 1995;11(1):31–6. – *Full text not available*
52. Iwasaki N, Minami A. Arthroscopically assisted reattachment of avulsed triangular fibrocartilage complex to the fovea of the ulnar head. J Hand Surg Am. 2009;34(7):1323–6. – *Surgical note and short-term follow-up of an already included study*
53. Haugstvedt JR, Husby T. Results of repair of peripheral tears in the triangular fibrocartilage complex using an arthroscopic suture technique. Scand J Plast Reconstr Surg Hand Surg. 1999;33(4):439–47- *No rehabilitation protocol*.
54. Gerlach D, Chun K, Trumble T, Gerlach DJ, Chun KF, Trumble TE. Triangular Fibrocartilage Complex Repair Through Bone Tunnels (Palmer Type 1D). OPERATIVE TECHNIQUES IN SPORTS MEDICINE. 2010;18(3):173–80.- *Technical note*
55. Geller JS, Taormina DP, Greene JD, Dodds SD. Delayed Presentation of Unstable Triangular Fibrocartilage Complex Tears Treated with Volar Foveal Ligament Repair. J Wrist Surg. 2021;10(2):144–9. – *no outcome scores and rehab protocol*
56. Geissler W, Geissler WB. Arthroscopic Knotless Peripheral Ulnar-Sided TFCC Repair. JOURNAL OF WRIST SURGERY. 2015;4(2):143–7.- *Technical note*
57. Frank R, Slikker W, Al-Shihabi L, Wysocki R, Frank RM, Slikker W, et al. Arthroscopic-Assisted Outside-In Repair of Triangular Fibrocartilage Complex Tears. ARTHROSCOPY TECHNIQUES. 2015;4(5):E577–81*.- Technical note*
58. Fones L, Cole KP, Kwok M, Gallant GG, Tosti R. All-Inside Versus Outside-in Repair of Triangular Fibrocartilage Complex Peripheral Tears. Journal of Hand Surgery – *rehabilitation protocol not available*
59. Fellinger M, Peicha G, Seibert FJ, Grechenig W. Radial avulsion of the triangular fibrocartilage complex in acute wrist trauma: a new technique for arthroscopic repair. Arthroscopy. 1997;13(3):370–4.- *technical note*
60. Feitz R, van Kooij Y, van der Oest M, Souer J, Hovius S, Selles R, et al. Patient-Rated Wrist Evaluation Threshold for Successful Open Surgery of the Triangular Fibrocartilage Complex. JOURNAL OF WRIST SURGERY. 2023; - *outside scope of study*
61. Feitz R, Khoshnaw S, van der Oest M, Souer J, Slijper H, Hovius S, et al. Long-term patient-reported outcomes for open surgery of the triangular fibrocartilage complex. BONE & JOINT OPEN. 2021;2(11):981–7. – *no proper outcome measures, only questionaire*
62. Feitz R, van der Oest MJW, van der Heijden EPA, Slijper HP, Selles RW, Hovius SER. Patient-reported outcomes and function after reinsertion of the triangular fibrocartilage complex by open surgery. Bone Joint J. 2021;103(4):711–7- *associated fracture + concomitant surgeries*
63. Feitz R, Stip D, van der Oest M, Souer S, Hovius S, Selles R. Prognostic Factors in Open Triangular Fibrocartilage Complex (TFCC) Repair. J Hand Surg Glob Online. 2021;3(4):176–81. – *No outcome measures*
64. Farr S, Schüller M, Ganger R, Girsch W. Outcomes after Arthroscopic Debridement of the Triangular Fibrocartilage Complex in Adolescents. J Wrist Sur. 2018;7(1):43–50.- *No TFCC repair*
65. Farr S, Zechmann U, Ganger R, Girsch W, Farr S, Zechmann U, et al. Clinical experience with arthroscopically-assisted repair of peripheral triangular fibrocartilage complex tears in adolescents-technique and results. INTERNATIONAL ORTHOPAEDICS. 2015;39(8):1571–7.-*Each patient has different procedures done.*
66. Edgerton MT, Kollmorgen RC. A Novel All-Inside Arthroscopic Technique for Radial-Sided Triangular Fibrocartilage Complex Tears: A Case Report and Review of Literature. Hand (N Y). 2017;12(5):NP166–9. – *single case*
67. Dehghani M, Zareazadeh A, Ataei F, Dehghani S, Dehghani S. The effect of open surgery in comparison with supportive treatment on improvement of traumatic triangular fibrocartilage complex: a clinical trial study. Journal of isfahan medical school. 2021;38(598):818‐823*.- out of scope of study*
68. Corso SJ, Savoie FH, Geissler WB, Whipple TL, Jiminez W, Jenkins N. Arthroscopic repair of peripheral avulsions of the triangular fibrocartilage complex of the wrist: a multicenter study. Arthroscopy. 1997;13(1):78–84.- *No rehab protocol*
69. Cornu A, Amouyel T, Chantelot C, Saab M. Clinical, functional and prognostic results after repair of peripheral lesions of the triangular fibrocartilage complex: a retrospective study of 21 patients. Eur J Orthop Surg Traumatol. 2021;31(3):557–62. – *No rehabilitation+ associated fractures*
70. Cooney WP, Linscheid RL, Dobyns JH. Triangular fibrocartilage tears. J Hand Surg Am. 1994;19(1):143–54.- *no clinical follow up + bony procedures done.*
71. Clark NJ, Munaretto N, Ivanov D, Berger RA, Kakar S. Outcomes of ulnotriquetral split tear repair: a report of 96 patients. J Hand Surg Eur Vol. 2019;44(10):1036–40- *– no rehabilitation , additional bony procedures*
72. Choudhury M, Yap R, Chia D, Sajeev S, Alizada G, Jiang J, et al. Symptomatic Radial-Sided Tears of the Triangular Fibrocartilage Complex: An All-Arthroscopic Repair Using Bone Anchors. JOURNAL OF WRIST SURGERY. 2022; - *No outcome measures*
73. Choudhury MM, Yap RTJ, Chia DSY, Sajeev S, Jiang JKH. An All-arthroscopic Technique of Repair of Substance Tears of the Triangular Fibrocartilage Complex in Symptomatic Patients. Tech Hand Up Extrem Surg. 2023;27(3):169–74.- *Technical note*
74. Chou CH, Lee TS. Peripheral tears of triangular fibrocartilage complex: results of primary repair. Int Orthop. 2001;25(6):392–5. – *Associated fractures*
75. Cho CH, Lee YK, Sin HK. Arthroscopic direct repair for radial tear of the triangular fibrocartilage complex. Hand Surg. 2012;17(3):429–32. – *Single case*
76. Chen AC, Hsu KY, Chang CH, Chan YS. Arthroscopic suture repair of peripheral tears of triangular fibrocartilage complex using a volar portal. Arthroscopy. 2005;21(11):1406. – *Technical note*
77. Chen AC, Cheng YH, Chiu CH, Cheng CY, Chan YS. Functional Outcomes Are Similar After Arthroscopic Capsular Repair of Triangular Fibrocartilage Complex Tears Between Outside-In Technique and All-Inside Technique Using Pre-Tied Suture Device. Arthroscopy. 2022;38(5):1457–62*.-rehabilitation protocol is not complete*
78. Carratalá Baixauli V, Lucas García FJ, Martínez Andrade C, Carratalá Baixauli R, Guisasola Lerma E, Corella Montoya F. All-Arthroscopic Triangular Fibrocartilage Complex Ligamentoplasty for Chronic DRUJ Instability. Tech Hand Up Extrem Surg. 2019;23(1):44–51. -*reconstructive procedure*
79. Cardenas-Montemayor E, Hartl JF, Wolf MB, Leclère FM, Dreyhaupt J, Hahn P, et al. Subjective and objective results of arthroscopic debridement of ulnar-sided TFCC (Palmer type 1B) lesions with stable distal radio-ulnar joint. Arch Orthop Trauma Surg. 2013;133(2):287–93- *Only TFCC debridement*
80. Boquet J, Lefebvre B, Van Innis F. Clinical retrospective evaluation of the arthroscopic treatments of the triangular fibrocartilage complex of the wrist. Évaluation clinique rétrospective des traitements arthroscopiques des lésions du complexe triangulaire du poignet. 2012;31(2):91–6. – *not in English*
81. Binder A, Kerfant N, Wahegaonkar A, Tandara A, Mathoulin C, Binder AC, et al. Dorsal Wrist Capsular Tears in Association with Scapholunate Instability: Results of an Arthroscopic Dorsal Capsuloplasty. JOURNAL OF WRIST SURGERY. 2013;2(2):160–7.- *associated Scapholunate Instability*
82. Bayoumy MA, El-Sayed A, Elkady HA, Saleh WR, Said HG, Ali AM. Arthroscopic Treatment of Type 1B Triangular Fibrocartilage Complex Tear by “Outside-In” Repair Technique Using Transcapsular Transverse Mattress Suture. Arthroscopy Tech. 2017;6(5):e1581–6.- *Surgical note*
83. Baratz ME. Central TFCC Tears in Baseball Players. Hand Clin. 2012;28(3):339- *review article without patient study base*
84. Auzias P, Camus E, Moungondo F, Van Overstraeten L, Auzias P, Camus EJ, et al. Arthroscopic-assisted 6U approach for foveal reattachment of triangular fibrocartilage complex with an anchor: Clinical and radiographic outcomes at 4 years’ mean follow-up. HAND SURGERY & REHABILITATION. 2020;39(3):193–200*.- No rehabilitation + associated with fractures*
85. Atzei A, Tandioy-Delgado FA, Marcovici LL, Aust TW, Luchetti R. Allinside Anatomic Arthroscopic (3A) Reconstruction of Irreparable TFCC Tear. J Wrist Sur. 2023;12(1):2–8. – *Cases of TFCC reconstruction*
86. Atzei A, Rizzo A, Luchetti R, Fairplay T. Arthroscopic foveal repair of triangular fibrocartilage complex peripheral lesion with distal radioulnar joint instability. Techniques in Hand and Upper Extremity Surgery. 2008;12(4):226–35.- *Paper on surgical Technique*
87. Atzei A, Luchetti R, Garagnani L, Atzei A, Luchetti R, Garagnani L. Classification of ulnar triangular fibrocartilage complex tears. A treatment algorithm for Palmer type IB tears. JOURNAL OF HAND SURGERY-EUROPEAN VOLUME. 2017;42(4):405–14.- *review article*
88. Armillotta L, Pogliacomi F, Corradi M, Ceccarelli F. Traumatic triangular fibrocartilage complex tears in the athlete: Results after arthroscopic treatment. J Orthop Traumatol. 2012;13:S123. – *includes cases of TFCC debridement only*
89. Argintar E, Mantovani G, Pavan A. TFCC reattachment after traumatic DRUJ instability: A simple alternative to arthroscopic management. Techniques in Hand and Upper Extremity Surgery. 2010;14(4):226–9.- *Associated with distal end radius fracture*
90. Anderson ML, Larson AN, Moran SL, Cooney WP, Amrami KK, Berger RA. Clinical comparison of arthroscopic versus open repair of triangular fibrocartilage complex tears. J Hand Surg Am. 2008;33(5):675–82.-*No immobilization protocol, no splints*

**Appendix III: Data extraction instrument**

| Sl. No | Study | |
| --- | --- | --- |
| 1 | Title |  |
| 2 | Author/s |  |
| 3 | Year |  |
| 4 | Type of study |  |
| 5 | Publication year |  |
| 6 | No of cases |  |
| 7 | Mean Age |  |
| 8 | Male: Female |  |
| 9 | Type of tear |  |
| 10 | Mean duration between injury to surgery |  |
| 11 | Type of surgery |  |
| 12 | Mean follow-up duration |  |
| 13 | Period and type of complete immobilization |  |
| 14 | Type of removable splint |  |
| 15 | Commencement of elbow ROM |  |
| 16 | Commencement of forearm ROM |  |
| 17 | Commencement of wrist ROM |  |
| 18 | Strengthening exercises |  |
| 19 | Outcome measures and post-op score |  |
| 20 | Grip strength |  |
| 21 | Return to activity |  |
